# Inhibition of Delta-like Ligand 4 enhances the radiosensitivity and inhibits migration in cervical cancer via the reversion of epithelial–mesenchymal transition

**DOI:** 10.1186/s12935-020-01434-1

**Published:** 2020-07-28

**Authors:** Shan-Shan Yang, De-Yang Yu, Yu-Ting Du, Le Wang, Lina Gu, Yun-Yan Zhang, Min Xiao

**Affiliations:** 1 Department of Gynecological Radiotherapy, Harbin Medical University Cancer Hospital, No. 150 HaPing Road, Nangang District, Harbin, 150081 China; 2 Department of Radiation Physics, Harbin Medical University Cancer Hospital, Harbin, 150081 China; 3 Department of Breast Surgery, Harbin Medical University Cancer Hospital, No. 150 HaPing Road, Nangang District, Harbin, 150081 China

**Keywords:** DLL4, Cervical cancer, Radiosensitivity, Progression, Epithelial–mesenchymal transition

## Abstract

**Background:**

Concurrent chemoradiotherapy is the common first-line treatment for patients with advanced cervical cancer. However, radioresistance remains a major clinical challenge, which results in recurrence and poor survival. Many studies have shown the potential of Delta-like Ligand 4 (DLL4) as a novel prognostic biomarker and therapeutic target in many solid tumors. Previously, we have found that high DLL4 expression in tumor cells may predict the pelvic lymph node metastasis and poor prognosis in patients with cervical cancer. In our present study, we further studied the effects of DLL4 on the biological behavior and radiosensitivity of cervical cancer cells.

**Methods:**

The expression of DLL4 and epithelial–mesenchymal transition (EMT) phenotype markers in cervical cancer cell lines or tissues were detected using Western blotting, and the expression of DLL4 mRNA in cervical cancer cell lines or tissues was detected using Quantitative real-time PCR. The effect of DLL4 on cell proliferation, migration, and radiosensitivity was evaluated using the CCK8 assay, flow cytometry, Transwell assays for cell invasion and migration, and Immunofluorescence staining in vitro.

**Results:**

The expression of DLL4 in radiotherapy-resistant SiHa cells was significantly higher than that in radiotherapy-sensitive Me-180 cells. Furthermore, downregulation of DLL4 enhanced the radiosensitivity of SiHa and Caski cells via the inhibition of cell proliferation, promotion of radiation-induced apoptosis, and inhibition of the DNA damage repair. Moreover, downregulation of DLL4 inhibited the EMT and reduced the proliferation, invasion, and migration ability in SiHa and Caski cells. Consistent with the DLL4 expression in the cell lines, the expression of DLL4 in the tissues of the radioresistant group was also higher than that of the radiosensitive group.

**Conclusions:**

Downregulation of DLL4 inhibited the progression and increased the radiosensitivity in cervical cancer cells by reversing EMT. These results indicated the promising prospect of DLL4 against the radioresistance and metastasis of cervical cancer and its potential as a predictive biomarker for radiosensitivity and prognosis in patients with cervical cancer patients receiving concurrent chemoradiotherapy (cCRT).

## Background

Cervical cancer (CC) ranks as the fourth most common malignancy and also the fourth most frequent cause of cancer-related mortality in women worldwide [1]. Clinically, radiation therapy (RT) serves as the common treatment of CC therapy management, RT is the preferred treatment for advanced-stage CC, and the main means of postoperative adjuvant therapy for early-stage CC. More than 60% of CC patients receive RT [2]. Nevertheless, acquired radioresistance leads to local therapeutic failure and poor outcomes in patients with CC. Thus, exploring the effective and reliable biomarkers for early prediction of radiosensitivity and the mechanisms underlying radioresistance in CC is important.

Epithelial–mesenchymal transition (EMT) is a fundamental biological process during which tumor cells develop more invasive phenotypes through the loss of epithelial characteristics and acquisition of mesenchymal characteristics [3]. This process plays an important role in metastasis of many cancers [4]. The roles of EMT is in cell invasion and migration, tumor recurrence, and chemotherapy resistance have been investigated intensively [5–9]. Recent emerging evidence suggests that EMT also plays a determinant role in the development of RT resistance of cancer cells [10–13]. In CC, the emergence of EMT is associated with the expression of some proliferative and proangiogenic proteins and implies the poor prognosis of locally advanced CC [14]. However, the role of EMT in the development of CC radioresistance remains unclear.

We previously found that high DLL4 expression level in tumor cells may predict the pelvic lymph node metastasis and poor survival in patients with FIGO stages I–II cervical cancer [15]. DLL4 is a novel transmembranous Notch ligand and shows a key function in angiogenesis during embryonic and postnatal development [16]. DLL4 blockade has been shown to inhibit tumor growth and angiogenesis in some murine tumor models [17]. and can prevent metastasis by inhibiting EMT, decreasing the number of cancer stem cells, and circulating tumor cells in Lewis lung Carcinoma [18]. Therefore, our present study aims to vertify whether DLL4 blockade can enhance the radiosensitivity, reduce EMT phenotypes in CC cells, and provide a potential therapeutic target for the sensitization of CC cells.

## Materials and methods

### Patients and tissue sample

Ten pairs of fresh CC tissues from the radiosensitive group and radioresistant groups were obtained from the FIGO stages III patients with CC who underwent concurrent chemoradiotherapy (cCRT) at the Department of Gynecological Radiotherapy, Harbin Medical University Cancer Hospital, between January 1, 2018 and January 1, 2019. All the patients were diagnosed with squamous CC through biopsy without history of other malignancy, and no patient received chemotherapy, RT, or immunotherapy before cCRT. The Clinicopathological characteristics of these patients are shown in Table 1.

**Table 1:** Clinicopathological characteristics of the patients with cervical squamous cell carcinoma in the RT-sensitive group (CC-RS) and RT-resistant (CC-RR) group

Variables	No. patients (*N *= 20)	Radiosensitivity	
		CC-RS (N = 10)	CC-RR (N = 10)
Age (years)			
< 45	13	6 (46.2)	7 (53.8)
≥ 45	7	4 (57.1)	3 (42.9)
FIGO stage			
IIIa	8	3 (37.5)	5 (62.5)
IIIb	12	7 (58.3)	5 (41.7)
SCC-Ag value			
1.5–10	15	7 (46.7)	8 (53.3)
> 10	5	3 (60.0)	2 (40.0)
Tumor size(cm)			
≤ 4	14	6 (42.9)	8 (57.1)
> 4	6	4 (66.7)	2 (33.3)
Deep stromal invasion			
No	5	3 (60.0)	2 (40.0)
Yes	15	7 (46.7)	8 (53.3)
Pelvic lymph node metastasis			
No	12	7 (58.3)	5 (41.7)
Yes	8	3 (37.5)	5 (62.5)

*CC*-*RS* cervical cancer patients in RT-sensitive group, *CC*-*RR* cervical cancer patients in RT-resistant group, *FIGO* Federation International of Gynecology and Obstetrics, *SCC* squamous cell carcinoma

The RT sensitivity was determined through the tumor response according to Response Evaluation Criteria in Solid Tumors (RECIST v1.1.). The therapeutic outcome was evaluated 6 to 8 weeks after cCRT completion on the basis of pelvic CT or MRI examination. Treatment response was defined as follows: complete response (CR): disappearance of all lesions; partial response (PR): ≥ 30% shrinkage in the sum of lesion size; progressive disease (PD): ≥ 20% increase in the sum of lesion size or appearance of one or more new lesions; and stable disease (SD): neither sufficient shrinkage to qualify for PR nor sufficient increase to qualify for PD. Patients with CR and PR were defined as RT-sensitive (CC-RS), whereas those patients with SD and PD were defined as RT-resistant (CC-RR).

This study complied with the Helsinki Declaration and was approved by the Ethics Committee of Harbin Medical University Cancer Hospital (Harbin, China). All patients provided their informed consents.

### Cell lines and tissue culture

The CC cell lines (i.e., SiHa, Caski, Me180 and C33A) were obtained from the cell bank of the committee on Type Culture Collection of the Chinese Academy of Sciences (Shanghai, China). Caski cells were cultured with RPMI-1640 medium, the SiHa and C33A cells were cultured with Modified Eagle’s Medium, and the Me180 cells were cultured with McCOY’s 5A medium. All media were supplemented with 100 unit/ml of penicillin, 100 mg/ml of streptomycin (Gibco, Life Technologies Inc., Grand Island, NY), and 10% fetal bovine serum (FBS). All cell lines were cultured in a humidified incubator maintained at 5% CO_2_ and 37 °C.

### RNA extraction and real time-PCR

Total RNA was extracted using TRIzol Reagent (Invitrogen), and the RNA was quantified using spectrophotometry (NanoDrop 2000, Thermo Fisher Scientific). The cDNAs were synthesized using the Verso cDNA kit (Thermo Fisher Scientific) in accordance with the manufacturer’s instructions. Real-time PCR assay was performed in triplicate using the SYBR-Green PCR Master kit (Applied Biosystems) and the ABI 7500 Fast Real-time system (Applied Biosystems). Primers to DLL4 were as follows: forward, 5′-AACTACTGCACCCACCACTCC-3′; reverse, 5′-GCCATCCTCCTGGTCCTTACA-3′. β-Actin genes were used to detect the normalization of each sample, and its primers were as follows: forward, 5′-CTTAGTTGCGTTACACCCTTTCTTG-3′; reverse, 5′-CTGTCACCTTCACCGTTCCAGTTT-3′. The PCR amplification conditions were as follows: 95 °C for 10 min, and 40 cycles of 95 °C for 10 s, 60 °C for 20 s, and 72 °C for 30 s. Results were calculated using the 2^−△△C(t)^ method [19].

### Western blot analysis

The total proteins were extracted from the cultured CC cells and frozen tissues CC tissues using the RIPA buffer supplemented with Halt Protease and Phosphatase Inhibitor Cocktail (100×, 78440, Thermo Fisher Scientific). The supernatant was obtained by centrifuging the insoluble material at 12,000 rcf for 20 min at 4 °C. The protein concentration was detected using BCA Protein Assay Kit (Pierce Biotechnology), and the samples were denatured at 95 °C for 5 min. Then, 30 μg of the extracted proteins were isolated via sodium dodecyl sulfate–polyacrylamide gel electrophoresis and transferred onto PVDF membranes (Millipore Company). The membranes were incubated with 5% nonfat dry milk in TBS-T at room temperature for 1 h and subsequently incubated with the primary antibodies supplemented with 5% nonfat dry milk in TBS-T overnight at 4 °C. The blots for glyceraldehyde-3-phosphate dehydrogenase (GAPDH) (1:4000) (sc-32233, Santa Cruz) served as the normalized control. After washing the membranes with TBS-T, the membranes were incubated with horseradish peroxidase-conjugated secondary antibody for 1 h at room temperature and washed again. The immunoreactive bands were then visualized using the ECL reagent (Seven Seas).

The primary antibodies were as follows: DLL4 rabbit polyclonal antibody (1:1000, ab7280, Abcam), PARP (1:500, # 9542S, Cell Signaling Technology), E-cadherin (1:1000, TA800670, ZSGB-BIO), N-cadherin (1:1000, TA503775, ZSGB-BIO), vimentin (1:1000, TA801297, ZSGB-BIO), Snail1(1:500, TA506430, ZSGB-BIO), Zeb1(1:1000, TA802313, ZSGB-BIO), MMP-2(1:1000, TA309752, ZSGB-BIO), MMP-9 (1:500, TA336901, ZSGB-BIO).

### RNA interference and transfection

The DLL4 small interfering RNA 1 (siRNA1) (SASI_Hs02_00352665) and DLL4 siRNA2 (SASI_Hs01_00174509) were obtained from Sigma-Aldrich, and the negative control siRNA was obtained from Invitrogen (12935-200). The sequence of the SiRNAs was as follows: DLL4 siRNA1 is sense (5′–3′) GUGACAAGAGCUUAGGAGA (dTdT), antisense (5′–3′) UCUCCUAAGCUCUUGUCAC (dTdT); and DLL4 siRNA2 is sense (5′–3′) GUCAUUGCCACGGAGGUAU (dTdT), antisense (5′–3′) AUACCUCCGUGGCAAUGAC (dTdT). Caski and SiHa cells were seeded in 6-well plates and incubated at 37 °C in a humidifed incubator. Lipofectamine RNAiMAX transfection reagent (Invitrogen) was used to transiently transfect the cells with 40 nM siRNA in accordance with the manufacturer’s instructions when the confluence was 60–80%. After 6–8 h, the medium was absorbed, and the incubation was continued using the complete medium.

### Cell proliferation assay

The cell counting kit-8 (CCK8) assay was performed to determine the CC cell proliferation. In brief, 12 h after transfection, the cells were seeded at a density of 1500 cells/well into 96-well culture plates and incubated for 24, 48, and 72 h. Then, the CCK-8 solutions were added and incubated in accordance with the manufacturer’s protocol. The absorbance was measured at 450 nm using a microplate reader (Thermo Fisher Scientific), and then the proliferation rate was calculated. For the radiosensitivity detection, the density of the cell seeding was 8000 cells/well, and the cells were irradiated at 0, 2, 4, 6, and 8 Gy of high-energy X-ray using a linear accelerator after 24 h of incubation. The time points of detection were 24 h and 48 h.

### Flow cytometric analysis

After transfection and incubation for 24 h, cells were irradiated with high-energy X-ray at 8 Gy and cultured at 37 °C for 48 h. For the cell cycle assay, the cells were harvested and fixed with ice-cold 70% ethanol. After incubating at − 20 °C overnight, the cells were centrifuged them at 2000 rpm for 5 min. Then, the supernatant was discarded, and 500 μl of 1× PBS was added to the suspended cells. The cells were labeled with 2.5 μg DAPI and incubated in dark for 20 min. Finally, the cell cycle was detected using flow cytometry (FACS Calibur, BD Biosciences). For the apoptosis assay, we cells collected and washed the cells with cold PBS twice. After centrifugation, the cells were resuspended with 90 μl of 1× binding buffer, and stained with 5 μl Annexin V-FITC and 5 μl PI for 15 min in dark using an Annexin V-FITC apoptosis detection kit (BD Bioscience, Oxford, UK). Then, 400 μl of 1× binding buffer was added to the cells before detection, and all specimens were subjected into flow cytometry (FAC Calibur) to detect cell apoptosis.

### Transwell assay for cell invasion and migration

The migration or invasion Transwell assays were conducted using 24-well Gelatin- or Matrigel-coated Transwell chambers (8-μm pore size, Corning Coster). At 24 h after transfection, the cells were seeded into the upper chamber and cultured with OPTI-MEM (Gibco) without FBS in a humidified incubator at 37 °C. For the migration assay, the seeding density was 1 × 10^5^ cells/well. For invasion assay, the seeding density was 0.75 × 10^5^ cells/well. The bottom chamber contained the complete medium containing 20% FBS. After migration for 12 h or invasion for 24 h, the filters were rinsed with PBS and fixed in the Hema3^®^ fixative (Fisher Scientific) for 30 min. The non-invaded/non-migrated cells in the upper chamber were removed gently, and the filters were stained with the Hema3^®^ solution I and II for 30 min in accordance with the manufacturer’s instructions. Lastly, the filters were mounted onto the slides with gridded coverslips and counted under an optical microscope (OLYMPUS IX51).

### Immunofluorescence staining

Cells were seeded at the density of 10 × 10^4^ cells/well onto covered slips which placed in six-well plates and incubated for 12 h. After that, the cells were exposed to 8 Gy high energy X-ray, and then put back to incubate for 24 h. Then, the cells were fixed in 4% paraformaldehyde (Solarbio) for 20 min, incubated in PBS supplemented with 0.2% Triton X-100 (Solarbio) for 10 min, and blocked in PBS supplemented with 10%FBS and 1% bovine serum albumin for 1 h at room temperature in sequence. Thereafter, the cells were incubated with rabbit monoclonal antibody gammaH2AX (γ-H2AX) (Ser139, Cell Signaling Technology, 1:400), E-cadherin (WL00941, Wanleibio, 1:200), or Vimentin (WL01960, Wanleibio, 1:200) diluted in PBS/10%FBS + 1%bovine serum albumin at 4 °C overnight. The next day, after rinsing with PBS, the cells were incubated with Alexa Fluor^®^ 488 conjugated, goat anti-rabbit IgG (H + L) (Thermo Scientific) at 1:400 for 1 h at 37 °C in dark. Hoechst 33342 (1:10,000, Molecular Probes) was used for nuclear counterstaining. Slides were mounted using ProLong Diamond Antifade Mountant (#P36965, Invitrogen).

### Statistical analysis

All data were presented as mean ± SD of at least three different experiments, and statistical analysis was performed using unpaired Student’s t-test or one-way analysis of variance through the SPSS 16.0 for Windows (SPSS, Chicago, USA). *P *< 0.05 was considered statistically significant.

## Results

### Efficiency of DLL4 knockdown in CC cells

**Fig. 1:**
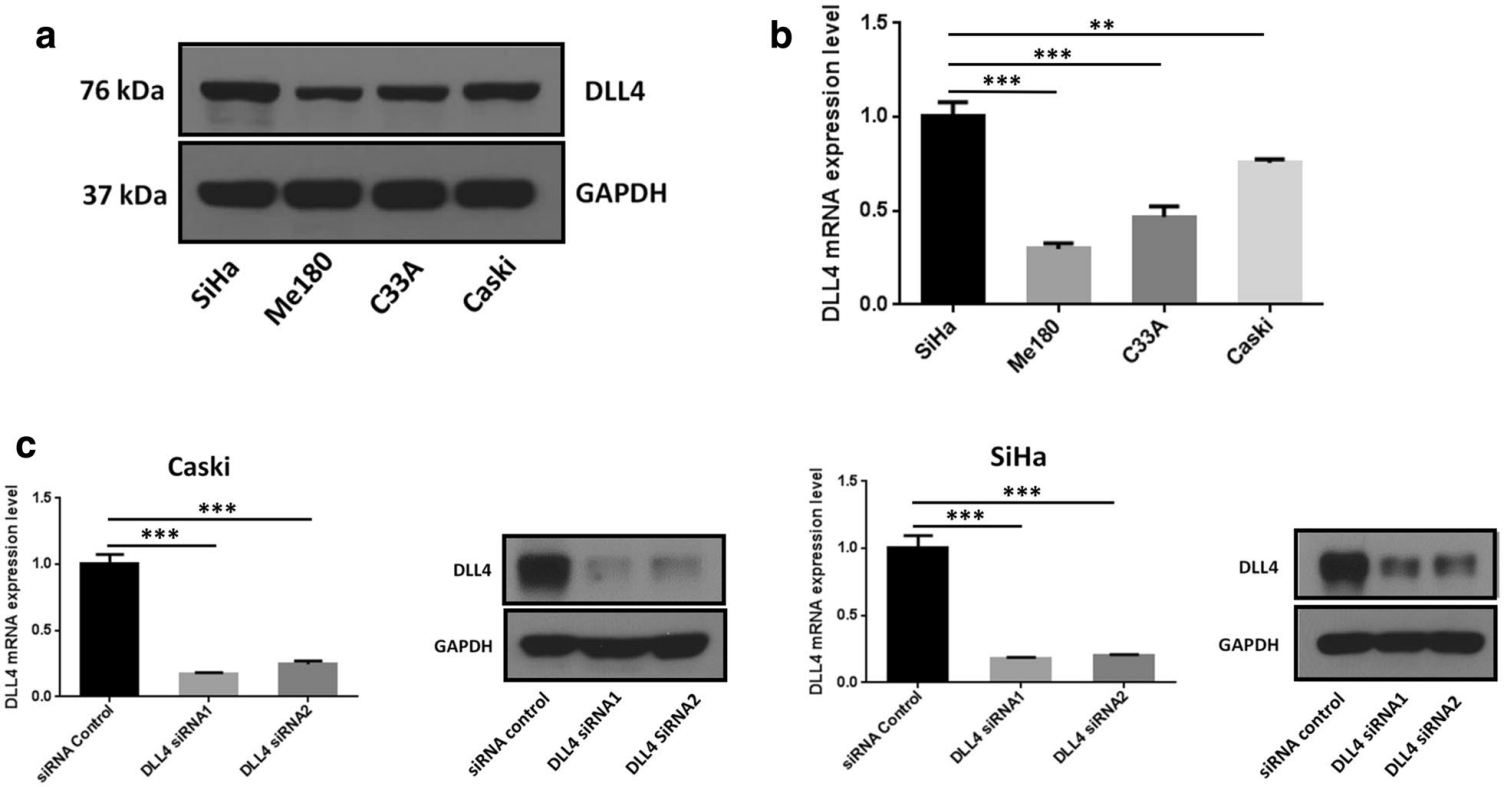
Efficiency of DLL4 knockdown in cervical cancer cells. **a** Western blot analysis and **b** real-time PCR analyses of the DLL4 expression levels in SiHa, Me180, C33A, and Caski CC cells at mRNA and protein levels. **c** Real‑time PCR and Western blot analyses was performed to detect the silencing efficiency of the two siRNA sequences, expression level of DLL4 was significantly downregulated in the DLL4 siRNA groups compared with the control group at both the mRNA and protein levels in Caski and SiHa cells. **P < 0.01, ***P < 0.001

Real-time PCR and Western blot were performed to detect the expression levels of DLL4 mRNA and protein in four CC cell lines (i.e., HeLa, SiHa, Caski and C33A). As shown in Fig. 1a, b, the relative expression level of DLL4 followed the order: SiHa > Caski > C33A > Me180. The RT-insensitive SiHa cells and the RT-sensitive Me-180 cells are the cell models widely used in the RT sensitivity study of CC [20]. Results showed that DLL4 was highly expressed in the SiHa cells but lowly expressed in the Me-180 cells.

The SiHa and Caski cells with high DLL4 expression were selected to knock down DLL4 using the DLL4 siRNAs. At 48 h after the transfection, the expression level of DLL4 was evidently decreased in SiHa and Caski cells (Fig. 1c). These results indicated that DLL4 was knocked down successfully in these two CC cell lines.

### DLL4 knockdown induced cell apoptosis, and inhibited cell proliferation, migration and invasion in CC cells

**Fig. 2:**
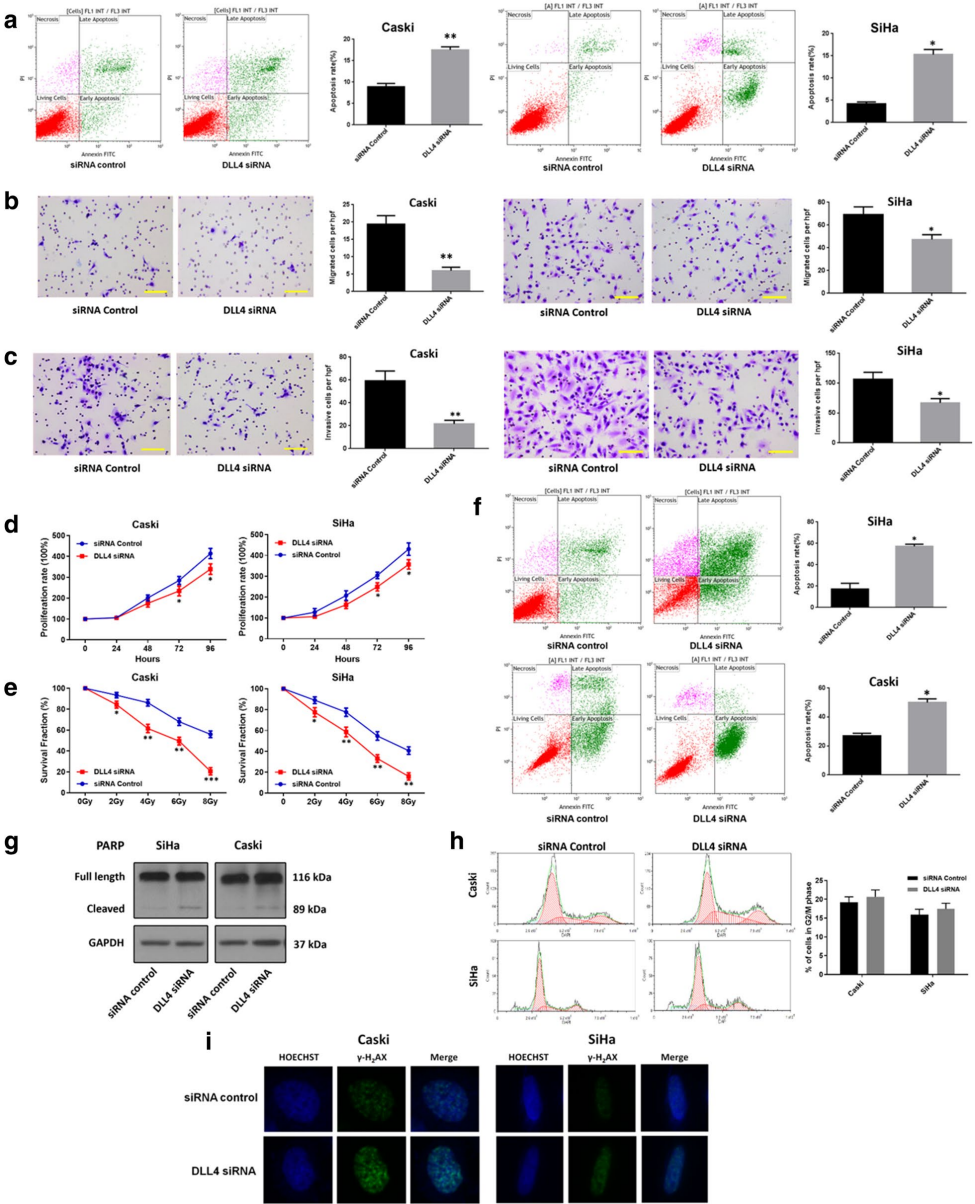
DLL4 downregulation induces cell apoptosis, inhibits cell proliferation, migration and invasion, increased the radiosensitivity in the cervical cancer cells. **a**, **f** Annexin V/propidium iodide (PI) double-staining apoptosis assay showed that DLL4 downregulation induced cell apoptosis and stimulated radiation-induced apoptosis significantly compared with the control group in Caski and SiHa cells. **b** Migration ability and **c** invasion ability of Caski and SiHa cells were detected using the Transwell assay. The migrated and invaded cells of the DLL4 siRNA groups were significantly less compared with those of the control group. **d**, **e** The CCK8 assay was performed to detect the proliferation ability of CC cells. DLL4 downregulation inhibited cell proliferation and induced radiation-induced cell death compared with the control group in Caski and SiHa cells. **g** Western blot was used to assess the effect of DLL4 downregulation on the expression level of full-length and cleaved PARPs in the irradiated Caski and SiHa cells. GAPDH was used as a loading control. **h** The Cell cycle analysis showed that DLL4 downregulation did not significantly stimulate radiation-induced G2/M cell cycle arrest compared with the vector controls in the irradiated Caski and SiHa cells. **i** γH2AX foci analysis by immunofluorescence was conducted to detect the function of DLL4 on the regulation of DNA damage repair. DLL4 downregulation increased the DNA double-strand breaks (DSBs) caused by irradiation, suggesting that the repair of DNA DSBs was delayed by DLL4-siRNA. *P < 0.05, **P < 0.01, ***P < 0.001

Flow cytometry was applied to detect whether DLL4 had an effect on the apoptosis of CC cells. In Caski cells, the apoptosis rate of the DLL4 siRNA group was (17.52% ± 0.708%) was significantly higher than that of the siRNA Control group (8.863% ± 0.805%), and the difference was statistically significant (*P *= 0.0002, Fig. 2a). A similar result was obtained in SiHa cells (*P *< 0.0001, Fig. 2a). Hence, DLL4 knockdown significantly induced the apoptosis of CC cells. The transwell assay results for cell invasion and migration showed that CC cells transfected with DLL4 siRNA acquired less migratory and invasive abilities compared with cells transfected with the siRNA control. In the Transwell migration assay, the number of SiHa cells that migrated through the gelatin-coated membrane in DLL4 siRNA group (47.33 ± 4.163) was significantly lower than that in the control group (69.00 ± 7.000) (*P *= 0.010, Fig. 2b). Similarly, the number of Caski cells that migrated through the gelatin-coated membrane in the DLL4 siRNA group (6.10 ± 1.00) was significantly lower than that in the control group (19.34 ± 2.157) (*P *= 0.001, Fig. 2b). In the Transwell invasion assay, the invasion ability of the SiHa-DLL4 siRNA cells (67.00 ± 7.211) was significantly lower compared with that of the control group (106.70 ± 11.520) (*P *= 0.0072, Fig. 2c). Similarly, the invasion ability of the Caski-DLL4 siRNA cells (21.67 ± 3.055) was significantly lower compared with that of the control group (59.33 ± 8.505) (*P *= 0.002, Fig. 2c). Therefore, results suggested that DLL4 knockdown inhibited the migration and invasion of the CC cells.

As to the proliferation ability, the CCK8 cell proliferation assay results demonstrated that the proliferation ability of CC cells in DLL4 siRNA group was inhibited significantly compared with the siRNA control group in Caski cells (72 h: *P *= 0.0432; 96 h: *P *= 0.0207) and SiHa cells (72 h: *P *= 0192; 96 h: *P *= 0.0263) (Fig. 2d).

### DLL4 knockdown enhanced the radiosensitivity of CC cells

The cell proliferation assay was performed to determine the effect of DLL4 knockdown on the radiosensitivity of CC cells. Contrary to the siRNA control group, the DLL4 siRNA group showed stronger sensitivity to RT from 2 Gy to 8 Gy (Fig. 2e) both in SiHa (2 Gy: *P *= 0.0213; 4 Gy: *P *= 0.0056; 6 Gy: *P *= 0.0024; 8 Gy: *P *= 0.0002) and Caski cells (2 Gy: *P *= 0.0193; 4 Gy: *P *= 0.0011; 6 Gy: *P *= 0.0033; 8 Gy: *P *= 0.0001), suggesting the increased radiosensitivity of CC cells after the downregulation of DLL4 expression.

### DLL4 knockdown induced apoptosis, but had no significant effect on the cell cycle in irradiated CC cells

Given that DLL4 knockdown inhibited the proliferation ability of irradiated CC, flow cytometric analyses were performed to study the effect of DLL4 knockdown on the cell cycle and apoptosis in irradiated CC cells.

The DLL4-siRNA group exhibited higher apoptosis rates of the SiHa and the Caski cells than the control group. At 24 h after irradiation at 6 Gy, the apoptosis rates for SiHa and Caski cells of the DLL4-siRNA groups were 50.01% ± 2.531% and 57.23% ± 1.983%, respectively, which were significantly higher than those in the control group (SiHa cells, 27.08% ± 1.643%; Caski cells, 17.10% ± 3.194%) (SiHa, *P *= 0.0002; Caski, *P* = 0.0003) (Fig. 2f). Furthermore, given that cleaved PARP is closely related to apoptosis, Western blot analysis was performed to detect the change in the expression of PARP (total and cleaved) after DLL4 knockdown in the irradiated SiHa and Caski cells. DLL4 knockdown upregulated the expression of cleaved PARP significantly (Fig. 2g). These results demonstrated that DLL4 knockdown can induce apoptosis, further verifying that the radiosensitivity of CC cells was improved after DLL4 knockdown.

In the cell cycle assay, DLL4 knockdown increased the percentage of cells in the G2/M phase in SiHa and Caski cells, but the increase was not statistically significant (Fig. 2h).

### DLL4 knockdown impaired the DNA damage repair in the irradiated CC cells

The effect of DLL4 on the regulation of DNA damage repair was detected by Immunofluorescence staining of the γH2AX foci. As shown in Fig. 2i, the DLL4 knockdown increased the DNA double-strand breaks (DSBs) caused by irradiation, suggesting that DLL4 knockdown delayed the repair of the DNA DSBs repair. Hence, radiosensitivity of CC cells by regulating the repair of DNA damage.

### DLL4 knockdown inhibited the EMT in CC cells

**Fig. 3:**
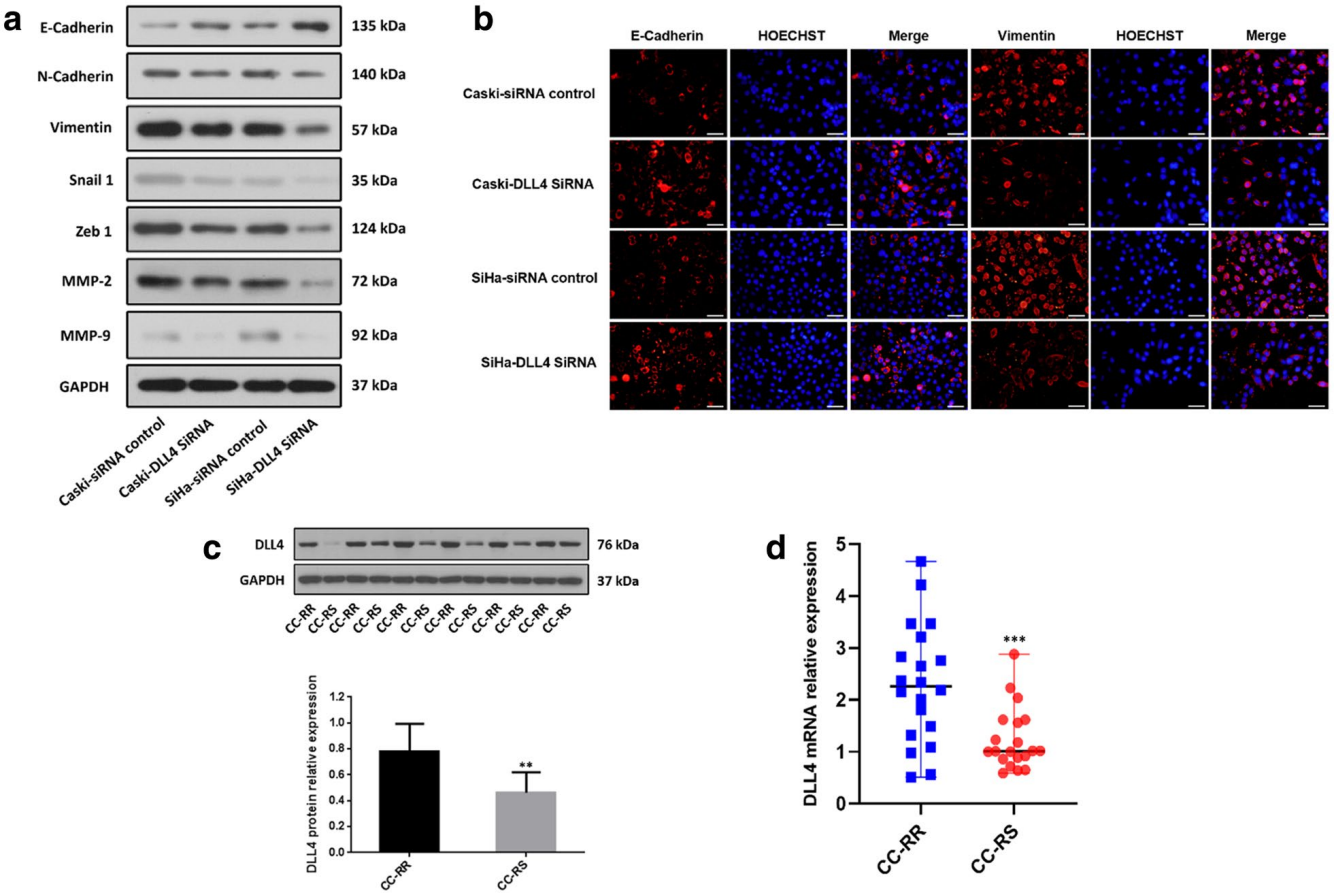
Western blot analysis demonstrated that DLL4 downregulation inhibits the EMT in the irradiated CC cells and DLL4 is upregulated in the CC-RR group. **a** Western blot analysis of the migration- and EMT-associated protein expression levels in the DLL4-siRNA group and control groups in the irradiated Caski and SiHa cells, GAPDH was used as the normalization control. **b** E-cadherin and vimentin analysis by immunofluorescence was conducted to detect the function of DLL4 on the regulation of EMT. **c** The DLL4 protein level detected by Western blot analysis and **d** the DLL4 mRNA level detected by Real-time PCR in radioresistance CC (CC-RR) group was significantly upregulated compared with that in radiosensitivity CC (CC-RS) group. The data are presented as mean ± SD, **P < 0.01, ***P < 0.001

EMT, as a biological process in which epithelial cells transform into mesenchymal phenotypic cells, not only enhances the ability of cell invasion and metastasis, and induces tumor cells to resist the killing effect of RT, chemotherapy and immunotherapy. Therefore, the correlation between the knockdown of DLL4 and the expression of EMT phenotype markers in the cell lysates of the cultured cells, was analyzed further. Knockdown of DLL4 in SiHa and Caski cells resulted in the upregulation of E-cadherin, which was the marker of the epithelial phenotype, and the downregulation of N-cadherin, vimentin, Snail1, and Zeb1, which were the markers of the mesenchymal phenotype, compared with the siRNA control group (Fig. 3a). The results of IF further verifed that DLL4 knockdown in SiHa and Caski cells resulted in the upregulation of E-cadherin and downregulation of vimentin, compared with the siRNA control group (Fig. 3b). DLL4 knockdown also resulted in the downregulation of the expression of MMP2 and MMP9 in the results of Westernblot (Fig. 3a). Therefore, DLL4 knockdown inhibited EMT and the secretion of MMP in CC cells.

### DLL4 was upregulated in CC tissues of radioresistant group (CC-RR)

The expression level of DLL4 in patients with cervical squamous cell carcinoma was detected in the CC-RR and the CC-RS groups using Western blot analysis and Real-time PCR to further certify the relationship between DLL4 and CC radiosensitivity. Results demonstrated that compared with the CC-RS group, the CC-RR group had significantly higher expression level of DLL4, both in protein level (*P *= 0.0012, Fig. 3c) and in mRNA level (*P *< 0.0001, Fig. 3d), further confirming our results in vitro.

## Discussion

Cervical cancer is a highly heterogeneous carcinoma with significant differences in radiosensitivity among patients [21]. The development of the intensity-modulated RT, as one of the main treatments for CC, is becoming increasing attractive [22, 23]. However, radioresistance, which limits the efficacy of RT and leads to local therapeutic failure-evoking recurrence, suggests a poor prognosis in RT-treated patients with CC. Therefore, the biological mechanism and related molecular biomarkers of the radiosensitivity in CC should be explored.

DLL4, as a main Notch ligand, is overexpressed in tumor vascular epithelial cells and tumor cells to activate the Notch pathway in many solid tumors [24]. This molecule plays a key role as a regulator for tumor angiogenesis, progression, and metastasis [25]. In our previous study, the DLL4 expression is closely related with the FIGO stage, lymphovascular space involvement, pelvic lymph node metastasis, and recurrence, predicting poor prognosis in patients with CC [15]. In current study, the effect of DLL4 on the biological behavior of CC was further studied, Results showed that DLL4 knockdown induced cell apoptosis, and inhibited cell proliferation, migration and invasion in vitro. In the studies of other cancers, DLL4 was also closely related to the invasion and metastasis ability of some tumor cells. In kidney cancer, Huang et al. have found that DLL4 overexpression increases the invasion and migration ability of kidney cancer cells through transwell and scratch assay, and leads to the considerable upregulation of the MMP2 and MMP9 expression [26]. The results from gastric cancer studies have also confirmed that the invasion and metastatic abilities of gastric cancer cells are significantly enhanced after the upregulated expression of DLL4 [27, 28].

To date, few studies have reported on the effect of DLL4 on the RT sensitivity of tumor cells. In human colon cancer xenografts, the Dll4 mAb can synergize the curative effect of ultrasound-stimulated microbubble and RT through a synergistic tumor growth delay of up to 24 days [29]. In our present study, the DLL4 expression RT-insensitive SiHa cells was upregulated compared that in RT-sensitive Me-180 cells. siRNAs were used to downregulate DLL4 and investigate the effect of DLL4 downregulation on the radiosensitivity of CC cells. Given that DLL4 was highly expressed in SiHa and Caski cells, siRNAs were used to downregulate DLL4 and the relevance between DLL4 and the radiosensitivity of CC cells was investigated. The CCK-8 assay indicated that DLL4 knockdown significantly decreased the proliferation rate compared with the control group in the irradiated CC cells. This result indicated that the downregulation of DLL4 enhanced the radiosensitivity of CC cells. In addition, the in vitro results showed that DLL4 knockdown enhanced the radiosensitivity of SiHa and Caski cells through the promotion of RT-induced apoptosis and inhibition of DNA damage repair but not via the cell cycle arrest. The relationship between DLL4 and radiosensitivity in CC was also proved in the Western blot analysis of patients receiving cCRT.

In the last decade, EMT has been widely recognized to play an important role in the progress and metastasis of tumor. In recent years, many studies have also suggested that EMT-like phenotype is related to the radioresistance of carcinoma cells via the inhibition of irradiation-induced cell death [30–32]. At the same time, EMT can induce the secretion of MMPs, leading to the degradation of the extracellular matrix, which further results in tumor cells escaping immune surveillance and developing radioresistance [33]. In the current study, the downregulation of DLL4 also upregulated the expression level of E-Cadherin and downregulated the expression levels of N-Cadherin, Vimentin, MMP2, and MMP9. These results indicated that DLL4 knockdown substantially inhibited the EMT in the CC cells, which may further lead to radioresistance.

## Conclusions

This study demonstrated that the DLL4 blockade may be a potential therapeutic target to overcome the radioresistance in CC cells and contribute to the inhibition of the progress and metastasis of CC. Furthermore, results suggested that DLL4 may be a biomarker to predict RT sensitivity of CC. However, a prospective study involving a high number of patients is warranted to validate the role of DLL4 as a novel predictive biomarker for the responses of cervical cancer patients with CC who are receiving cCRT. Further investigation is needed to elucidate the mechanism by which DLL4 mediates EMT and radiosensitivity and verify the feasibility of DLL4 as a clinical target.

## Data Availability

Not applicable.
